# Extended Oral Antibiotics After Primary Total Hip and Knee Arthroplasty in an Ambulatory Surgery Center: A Bayesian Threshold and Cost-Effectiveness Analysis

**DOI:** 10.1016/j.artd.2026.102108

**Published:** 2026-08-13

**Authors:** Breydan H. Wright, Charlotte C. Baker, Zachary M. Ricciardelli, Joseph M. Schwab, Thomas L. Bradbury, Courtney Levit, George N. Guild

**Affiliations:** a Total Joint Specialists, Advanced Center for Joint Surgery and Northside Hospital Forsyth, Cumming, GA, USA; b Medical College of Georgia, Augusta University, Augusta, GA, USA

**Keywords:** Total hip arthroplasty, Total knee arthroplasty, Extended oral antibiotics, Bayesian analysis, Cost-effectiveness

## Abstract

**Background:**

Given evidence for extended oral antibiotic prophylaxis (EOA), we compared EOA with no EOA after primary total hip and knee arthroplasty in an ambulatory surgery center (ASC) using Bayesian and probabilistic cost modeling.

**Methods:**

This retrospective study included same-day discharge THA/TKA cases at a single ASC. The primary outcome was 90-day periprosthetic joint infection (PJI). Bayesian logistic regression estimated posterior odds ratios (ORs) and probabilities of benefit. Probabilistic cost-effectiveness analysis incorporated antibiotic and debridement, antibiotics, and implant retention costs.

**Results:**

Among 7723 TKAs (EOA n = 2337; no-EOA n = 5386) and 5872 THAs (EOA n = 1670; no-EOA n = 4202), 90-day PJI rates were 0.13% versus 0.26% in TKA and 0.48% versus 0.40% in THA. Median posterior ORs were 0.58 for TKA (84.3% probability of benefit) and 1.24 for THA (30.7%). In TKA, the absolute risk reduction was 1.13 per 1000 cases, yielding a number needed to treat of 885;clinically favorable values would require baseline PJI rates of 0.8%-1.2%. With costs of $15 for EOA and $30,000 for PJI, EOA was cost-effective at $50,000 per PJI averted and cost-saving when PJI costs exceeded $13,000 in TKA, but not THA. Patient-reported outcome improvements were similar.

**Conclusions:**

In this low-risk, same-day discharge ASC cohort, EOA showed a modest probablitly of benefit and favorable economic performance in TKA. These findings support selective, risk-based use consistent with antibiotic stewardship.

## Introduction

Periprosthetic joint infection (PJI) remains one of the most serious complications following total hip and knee arthroplasty (THA and TKA), carrying significant morbidity, patient dissatisfaction, and substantial financial burden, with projected annual costs reaching $1.85 billion by 2030 [[1], [2], [3]]. Early postoperative infections pose a particular challenge in ambulatory pathways, where same-day discharge has become increasingly common for both THA and TKA [4].

Equally concerning are the psychosocial and mortality impacts PJI has on affected patients. Rates of depression are 4 times higher in those undergoing a 2-stage exchange for PJI than aseptic revisions [5]. Moreover, 2-stage exchange arthroplasty, the gold standard treatment for chronic PJI, is not without major mortality risk [6,7]. The overall survival of PJI following THA and TKA at 5 years has been demonstrated to be comparable or worse to several of the most common types of cancers [8,9]. In an effort to reduce PJI, extended oral antibiotics (EOA) have emerged as a potential solution; however, there is conflicting evidence on the value of EOA in PJI prevention. Several studies have supported the use of EOA to reduce early postoperative infection risk [10,11]. One study demonstrated benefit specifically among high-risk primary TKA and THA patients [12]. In contrast, 2 recent studies found no significant reduction in infection rates with EOA use in broader high-risk or standard-risk arthroplasty populations [13,14]. The rare event rate of early PJI further complicates traditional statistical inference and underscores the need for Bayesian methods that directly estimate the probability of benefit.

Cost implications also deserve attention. Although EOA introduces a modest pharmacy cost, preventing readmissions and DAIR procedures can make the approach cost-effective, or even cost-saving, in the right setting [15]. Notably, previous work has not incorporated probabilistic cost modeling specifically for ambulatory surgery center (ASC)-based primary THA/TKA populations.

Therefore, the purpose of this study was to evaluate the clinical and economic impact of EOA following primary THA/TKA in an ASC setting using Bayesian modeling. We hypothesized that Bayesian and decision-analytic modeling would identify infection-rate thresholds at which EOA becomes clinically and economically justifiable in ASC-based primary THA/TKA.

## Material and methods

With Institutional Review Board approval, a retrospective cohort study was conducted of consecutive primary THA/TKA procedures performed in an ASC from its start in August 2019 through May 2025, all managed with same-day discharge. Standard perioperative IV prophylaxis was used. Beginning in March 2023, eligible patients were prescribed EOA at discharge (cefdinir 300 mg twice daily for 7 days); patients with a documented cephalosporin anaphylaxis received doxycycline 100 mg twice daily for 7 days. Cefdinir was chosen for its relatively broad spectrum gram-positive and gram-negative coverage, which is characteristic of third-generation cephalosporins. Other than the EOA usage as described, no additional changes to the center's existing infection prevention protocols occurred during the study period.

### Cohorts and outcomes

Exposure was EOA vs no-EOA at discharge, and PJI was defined according to the 2013 Musculoskeletal Infection Society criteria [16]. Outcomes were evaluated by procedure type (THA vs TKA).

### Statistical analysis

Infection rates between the no-EOA and EOA groups were compared using Fisher exact tests. Frequentist estimates included observed event rates, risk ratios (RRs) with 95% confidence intervals (CIs), absolute risk differences, and number needed to treat/harm (NNT/NNH). We fit a Bayesian hierarchical logistic regression for PJI. Weakly informative priors were used (Normal [0, 1.5] on log-odds). Posterior odds ratios (OR) with 95% credible intervals (CrIs) and the posterior probability of benefit (OR < 1) were reported. Sensitivity analyses included propensity weighting and skeptical priors. NNT was calculated from both observed cohort data and Bayesian posterior expectations to distinguish observed effects from projected future benefit. A probabilistic cost-effectiveness analysis was conducted from the ASC perspective using assumed base–case values of $15 per patient for EOA prophylaxis and $30,000 per PJI requiring DAIR readmission, with results expressed as incremental cost-effectiveness ratios (ICERs) and probabilities of cost-effectiveness at a willingness-to-pay threshold of $50,000 per infection averted. Cost-saving thresholds were derived using the Bayesian expected absolute risk reduction, which differs from thresholds based on observed cohort rates. Expected cost per patient was calculated as the mean cost derived from probabilistic simulation, incorporating the probability of PJI, the cost of infection treatment, and the cost of EOA prophylaxis. Study cohort data obtained from Force Therapeutics (New York, NY), including demographic and patient-reported outcome measures, were summarized as means ± standard deviations or percentages, as appropriate (Tables 3). All analyses were conducted using R version 4.5.1 (Vienna, Austria).

**Table 1:** Baseline demographics and comorbidity profile by group.

Variable	No-EOA (n = 9586)	EOA (n = 4007)	*P* value
THA			
Age (y)	67.1 ± 10.7	66.2 ± 11.1	.003
Weight (kg)	84.6 ± 19.5	84.1 ± 19.9	.354
Height (cm)	171.5 ± 10.3	171.1 ± 10.5	.183
BMI (kg/m^2^)	28.6 ± 5.4	28.6 ± 5.7	.830
Gender			.444
F	55	56	
M	45	44	
Race			<.001
American Indian or Alaska Native	0.5	0.7	
Asian	0.6	0.5	
Black or African American	5.0	7.7	
Native Hawaiian or other Pacific Islander	0.2	0.3	
White	86	83	
Other	8.0	7.7	
Comorbidities			
Diabetes	6.8	8.1	.083
Hypertension	30	31	.686
High cholesterol	26	30	.006
Arthritis	48	48	.832
Heart disease	6.0	6.2	.698
Respiratory condition	4.1	4.6	.360
Depression	7.4	9.3	.010
Anxiety	12	15	<.001
Tobacco use	4.7	4.8	.571
Assistive device	15	21	.571
Narcotic use	10.0	9.6	>.999
TKA			
Age (y)	71.2 ± 8.7	69.6 ± 8.9	<.001
Weight (kg)	88.1 ± 18.0	90.2 ± 104.9	.302
Height (cm)	170.9 ± 10.6	170.1 ± 10.8	.002
BMI (kg/m^2^)	30.0 ± 5.0	31.2 ± 43.4	.184
Gender			.110
F	57	59	
M	43	41	
Race			<.001
American Indian or Alaska Native	0.5	0.3	
Asian	1.6	2.0	
Black or African American	3.6	6.3	
Native Hawaiian or other Pacific Islander	<0.1	0.1	
White	86	84	
Other	8.4	7.3	
Comorbidities			
Diabetes	11	13	.003
Hypertension	37	37	.528
High cholesterol	32	33	.842
Arthritis	57	57	.688
Heart disease	8.8	8.7	.836
Respiratory condition	5.1	4.8	.520
Depression	8.0	9.0	.117
Anxiety	12	14	<.001
Tobacco use	2.8	3.4	.073
Assistive device	11	19	<.001
Narcotic use	9.2	9.9	.106

F, female; M, male; BMI, body mass index.

Values are mean ± standard deviation or n (%).

**Table 2:** Data summary.

Group	EOA	No-EOA	Fisher’s exact *P* value	Observed absolute risk reduction	Observed number needed to treat/Harm	Risk ratio (RR, 95% CI)	Posterior probability of benefit (OR < 1)	Posterior expected absolute risk reduction	Posterior median NNT/NNH	Posterior OR (OR, 95% CrI)	Expected cost EOA	Expected cost no-EOA	Expected net savings per patient
THA	8/1670 (0.48%)	17/4202 (0.40%)	0.6619	−0.074%	NNH = 1342.8	1.184 (0.512-2.738)	30.7%	−0.097%	NNH = 1030.9	1.235 (0.523-2.706)	$254.52	$202.28	−$52.24
TKA	3/2337 (0.13%)	14/5386 (0.26%)	0.3041	0.132%	NNT = 760.1	0.494 (0.142-1.717)	84.3%	0.113%	NNT = 885.0	0.576 (0.157-1.633)	$79.18	$129.97	$50.78

**Table 3:** Patient-reported outcome measures by group.

Variable	No-EOA	EOA	*P* value
THA			
Procedure satisfaction (1-5, %)			.443
1	0.6	0.7	
2	1.1	1.4	
3	2.8	3.0	
4	13	14	
5	83	81	
Numeric Pain Rating Scale (NPRS 0-10, mean ± standard deviation)			
Preop	5.78 ± 2.00	5.80 ± 2.01	.728
Postop 6 wks	2.37 ± 1.50	2.55 ± 1.66	.002
Postop 12 wks	2.20 ± 1.61	2.33 ± 1.72	.216
TKA			
Procedure satisfaction (1-5, %)			.067
1	0.9	1.3	
2	1.1	1.7	
3	5.3	6.1	
4	22	22	
5	70	69	
Numeric Pain Rating Scale (NPRS 0-10, mean ± standard deviation)			
Preop	5.35 ± 2.03	5.36 ± 2.11	.877
Postop 6 wks	3.05 ± 1.66	3.18 ± 1.74	.008
Postop 12 wks	2.44 ± 1.54	2.61 ± 1.68	.008

### Infection prevention protocol

Institutional protocols adhered to existing guidelines for the prevention of PJI after primary hip and knee arthroplasty. Other than EOA, infection prevention protocols were identical between the study groups. Preoperatively, glycemic control was assessed by preoperative HbA1C in all patients with a surgical candidacy threshold of 7.5. Nicotine use was discouraged for 4 weeks before and 6 weeks after surgery. No surgery was performed within 3 months of an intra-articular steroid injection. Antirheumatic medications were managed in accordance with the 2022 American College of Rheumatology/American Academy of Hip and Knee Surgeons guidelines [17]. All patients underwent universal preoperative skin decolonization with chlorhexidine wipes the night before surgery. On arrival on the day of surgery, all patients underwent nasal decolonization with povidone iodine. Methicillin-resistant Staphylococcus aureus (MRSA) screening was not performed, but patients considered high risk (previous history of MRSA infection or physical proximity risk) were treated with chlorhexidine scrub showers and nasal mupirocin twice daily for 5 days before surgery. Two grams of intravenous cefazolin was administered within 1 hour of skin incision (3 g intravenous cefazolin for patients weighing >120 kg). High-risk MRSA patients received 1 g of intravenous vancomycin in addition to standard cefazolin dosing. The skin over the surgical site was treated with alcohol and a chlorhexidine-based skin preparation. The surgical field was isolated with adherent drapes and sealed with an iodophor-impregnated plastic adhesive incise drape. A dilute Betadine bath followed by irrigation with normal saline was performed before wound closure. Wounds were covered with sterile adhesive dressings without a planned dressing change for 7 days. Those in the EOA group started oral antibiotics in the morning on the day after surgery.

### Demographics

We analyzed 7723 TKAs (EOA n = 2337; no-EOA n = 5386) and 5872 THAs (EOA n = 1670; no-EOA n = 4202). Overall, the two groups were similar in terms of demographics and comorbidities (Table 1).

## Results

### Primary outcome: 90-day PJI

In this cohort of 13,593 total joint arthroplasties, overall infection rates were low, with 0.27% in the EOA group and 0.32% in the no-EOA group (Fisher's *P* = .736). Subgroup analyses for TKA showed an infection rate in the EOA group of 0.13% vs 0.26% in the no-EOA group (Fisher's *P* = .304), while THA infection rates were 0.48% in the EOA group and 0.40% in the no-EOA group (Fisher's *P* = .662) (Table 2).

### Frequentist analysis

In TKA, the lower infection rate with EOA (0.13% vs 0.26%) corresponded to an RR of 0.49 (95% CI 0.14-1.72), an absolute risk reduction of 0.132%, and an NNT of approximately 760. In contrast, THA showed a numerically higher infection rate with EOA (0.48% vs 0.40%), corresponding to an RR of 1.18 (95% CI 0.51-2.74), an absolute risk increase of 0.074%, and a number NNH of approximately 1343.

### Bayesian analysis

**Figure 1:**
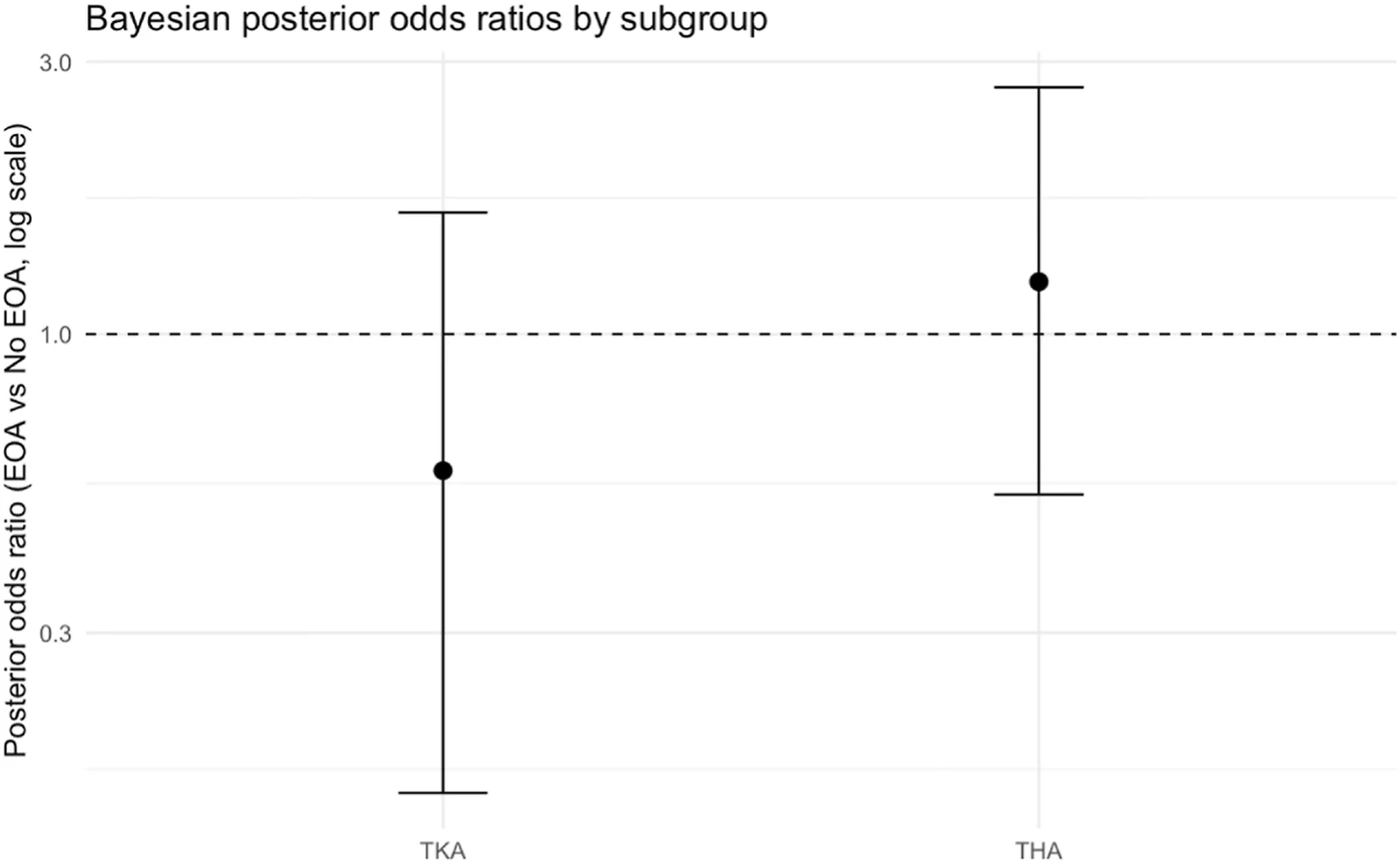
Bayesian posterior odds ratios for PJI associated with EOA vs no EOA, stratified by procedure type. Points represent posterior median odds ratios, and error bars indicate 95% credible intervals. The dashed horizontal line at 1.0 denotes no difference between groups. Values below 1.0 suggest a potential protective effect of EOA, whereas values above 1.0 suggest no benefit or possible harm. While the overall and TKA estimates trend toward benefit, credible intervals cross 1.0 in all subgroups, reflecting uncertainty in the estimated effect.

The TKA subgroup demonstrated an 84.3% posterior probability of benefit, with a median posterior OR of 0.58 (95% CrI 0.16-1.63) and a posterior median absolute risk reduction of 0.11%. In comparison, the THA subgroup demonstrated a lower probability of benefit (30.7%), with a median posterior OR of 1.24 (95% CrI 0.52-2.71) and a posterior median absolute risk difference of −0.10% (Fig. 1).

Posterior median NNT estimates reflected these findings, with a median NNT of approximately 751 in TKA, whereas THA demonstrated a median NNH of approximately 562.

### Number needed to treat analysis

We evaluated NNT as a function of baseline PJI risk using subgroup-specific Bayesian effect estimates. In TKA, a clinically acceptable NNT (≤200-300) would require baseline PJI rates of approximately 0.8%-1.2%. In contrast, THA demonstrated a low probability of benefit, and no clinically meaningful threshold for NNT was identified. These findings suggest that the potential clinical benefit of EOA increases as baseline infection risk approaches 1%, particularly in TKA.

### Distinction between observed and modeled NNT

The observed frequentist NNT reflects the absolute risk difference within the study cohort, whereas the Bayesian NNT represents the posterior expected benefit for future patients and incorporates uncertainty in treatment effect estimates.

### Cost-effectiveness analysis

Expected costs differed by procedure type. In TKA, EOA was associated with lower expected costs ($79.18 vs $129.97), corresponding to a net savings of $50.78 per patient, favoring EOA. In contrast, in THA, EOA was associated with higher expected costs ($254.52 vs $202.28), corresponding to a net increase of $52.24 per patient, favoring no EOA.

Across probabilistic simulations, EOA was cost-saving in 77.4% of iterations in TKA compared to 25.2% in THA, corresponding to net cost savings in TKA ($50.78 per patient) but increased costs in THA (–$52.24 per patient).

**Figure 2:**
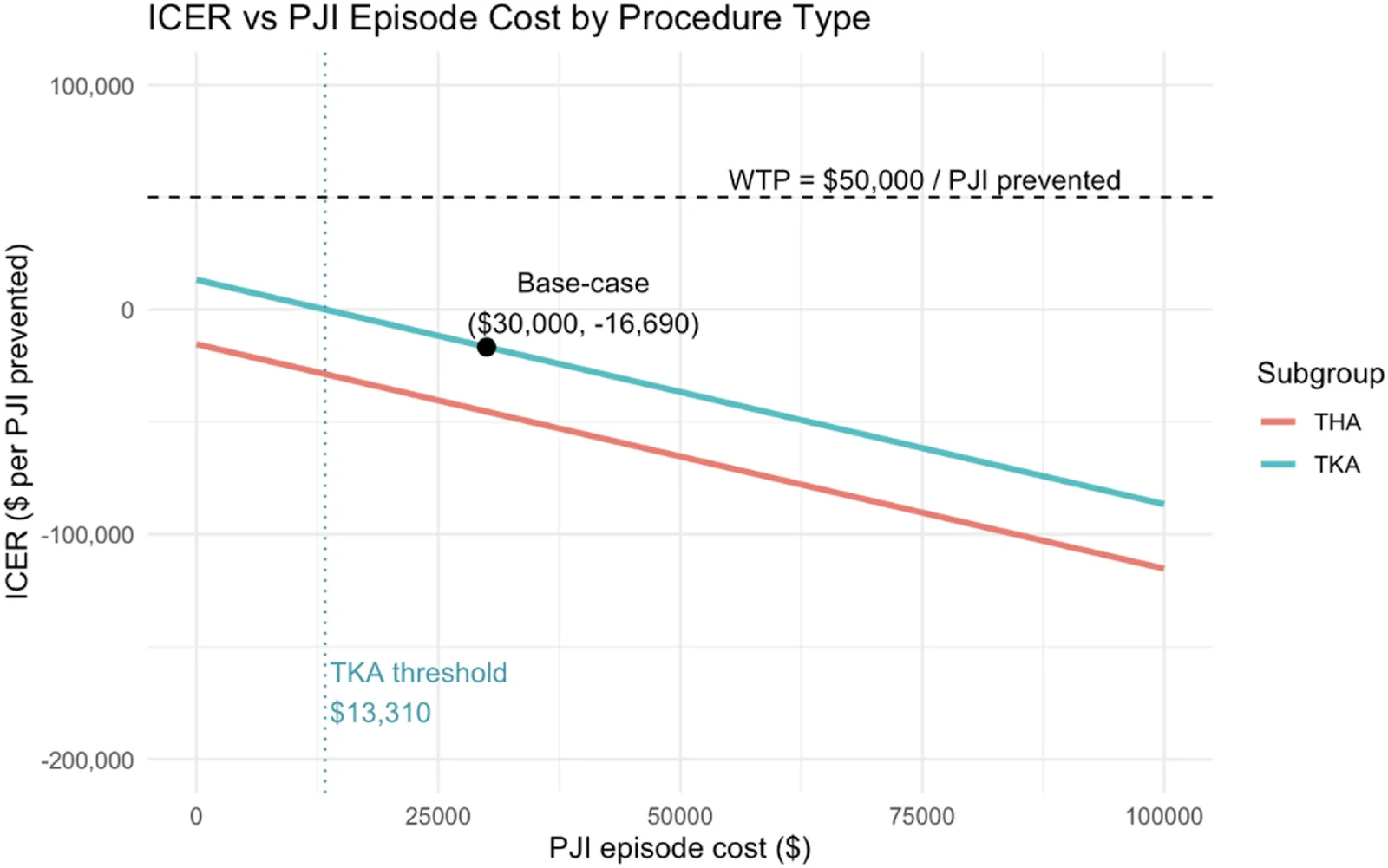
Incremental cost-effectiveness of EOA across a range of PJI episode costs, stratified by procedure type. Lines represent ICERs calculated using a Bayesian model–estimated absolute risk reduction for THA, and TKA. The horizontal dashed line indicates a willingness-to-pay (WTP) threshold of $50,000 per PJI prevented. Vertical dashed lines denote cost-saving thresholds, defined as the PJI episode cost at which EOA becomes cost-neutral (ICER = 0), estimated at approximately $13,310 for TKA; no cost-saving threshold was observed for THA due to a negative expected treatment effect. The base–case scenario assumes a PJI episode cost of $30,000. At this value, EOA is cost-effective but not cost-saving in the TKA cohort. Negative ICER values indicate cost savings.

The ICER demonstrated a monotonic relationship with PJI episode cost. EOA became cost-saving when the downstream cost of infection exceeded approximately $13,300 in TKA, whereas THA did not reach a cost-saving threshold within the evaluated range (Fig. 2).

### Demographics

Baseline demographic characteristics and comorbidity profiles were generally similar between the no-EOA and EOA cohorts, with comparable age, sex distribution, race, and prevalence of major medical comorbidities (Table 1). There were statistically significant differences between the no-EOA and EOA groups for THA in assistive device use and anxiety, and for TKA in diabetes, anxiety, and assistive device use.

### Patient-reported outcome measures

Patient-reported outcomes were similar between groups for both THA and TKA, with the majority of patients reporting the highest satisfaction score (5/5: THA, 83% no-EOA vs 81% EOA; TKA, 70% no-EOA vs 69% EOA) (Table 3). Pain scores improved from the preoperative period to the 12-week follow-up in both groups. In THA, a statistically significant difference was observed at 6 weeks postoperatively, while in TKA, differences were observed at both 6 and 12 weeks; however, these differences did not reach clinical significance.

## Discussion

In this large ASC cohort with routine same-day discharge, EOA after primary TJA were associated with a posterior probability greater than 50% of reducing early PJI risk in TKA, whereas THA demonstrated a lower probability of benefit. These procedure-specific findings likely reflect both the low overall event rate and underlying differences in infection risk between procedures, and suggest that the impact of extended antibiotic prophylaxis may not be uniform across arthroplasty types. Although the CrIs were wide, reflecting how rare these infections are, the Bayesian approach offers useful context by estimating the likelihood of benefit rather than depending solely on a binary significance test. These results are meaningful given the growing use of ASCs and the ongoing challenge posed by early postoperative infections [9,18]. While no universal threshold defines a “significant” effect in the Bayesian framework, posterior probabilities of approximately 0.95-0.975 are sometimes adopted as conventional analogs to frequentist significance. By this benchmark, the 84.3% posterior probability of benefit in the TKA subgroup represents suggestive but not definitive evidence, consistent with a 95% CrI that crosses the null. In rare-event prevention, posterior probabilities modestly exceeding equipoise (50%) may still meaningfully inform decision-making, particularly in low-cost, low-risk interventions where traditional frequentist thresholds may be overly conservative.

We observed baseline differences between groups, with the EOA cohort demonstrating higher rates of diabetes, assistive device use, anxiety, and depression. Diabetes, in particular, is a well-established risk factor for PJI. Importantly, receipt of EOA was determined by institutional protocol implementation at a defined time point rather than patient-level risk stratification, reducing concern for confounding by indication, although temporal confounding cannot be entirely excluded. Despite a higher prevalence of diabetes in the EOA cohort, the posterior probability still favored a reduction in the risk of early PJI.

Although the frequentist and Bayesian analyses are based on the same underlying data, they answer different clinical questions. Frequentist analyses evaluate whether the observed data are compatible with the null hypothesis and are often interpreted using arbitrary significance thresholds. In contrast, Bayesian analyses estimate the probability that a treatment is beneficial by combining the observed data with prespecified prior assumptions and explicitly accounting for uncertainty. This approach is particularly valuable in rare-event settings such as PJI, where low event rates often result in wide CIs and limited statistical power. Accordingly, the posterior probabilities reported in this study should be interpreted as evidence favoring or opposing benefit rather than definitive proof of efficacy, and should be considered alongside the corresponding CrIs and the broader clinical context.

The results align with prior work suggesting potential benefits of EOA in infection-prone populations [12,19]. Importantly, this analysis focuses on primary THA and TKA performed in an ASC setting, an area where infection–prevention strategies must balance safety with workflow efficiency, rapid discharge, and stewardship responsibilities. We found no signal of harm in short-term patient-reported outcomes.

Our findings also highlight an important contextual consideration for high-performing ASCs: when baseline infection rates are extremely low, the opportunity for any preventive intervention to demonstrate meaningful absolute benefit becomes inherently limited. In settings where PJI rates fall below 0.3%, even a truly effective strategy, such as EOA, yields only a small absolute risk reduction, resulting in NNTs that are impractically high and unlikely to influence individual patient outcomes. This phenomenon reflects the mathematical reality of prevention in low-risk environments rather than a failure of the intervention itself. Our threshold analysis further demonstrated that the potential clinical benefit of EOA increases as baseline infection rates approach approximately 1%, at which point the absolute benefit becomes more meaningful. Accordingly, our findings do not support routine universal EOA administration for standard-risk primary THA and TKA performed in low-risk ambulatory surgery centers. Rather, our findings are consistent with previous studies demonstrating benefit among selected high-risk primary arthroplasty patients and support a selective, risk-based approach to EOA rather than routine universal administration [12,19].

The cost analysis further supports clinical decision-making and highlights important procedure-specific differences. In TKA, EOA were frequently cost-effective across a range of willingness-to-pay thresholds and became cost-saving when the downstream cost of PJI exceeded approximately $13,300, which is lower than contemporary estimates of PJI treatment costs [2]. In contrast, EOA were not cost-saving in THA within the evaluated range. Given the high financial penalties associated with readmission and infection in value-based payment systems, these results have meaningful implications for health–system strategy [9]. The ICER-based framework complements Bayesian clinical analysis by contextualizing a modest probabilistic benefit within a cost structure relevant to contemporary value-based arthroplasty care. Notably, the divergence between probabilistic clinical benefit and cost-effectiveness reinforces that even small reductions in rare but costly complications can be economically justified.

### Antibiotic stewardship considerations

The potential benefit of extended oral antibiotic prophylaxis must be weighed against antibiotic stewardship principles [20]. Although the short, 7-day regimen used in this study represents a relatively small incremental antibiotic exposure, widespread routine use could contribute to unnecessary antibiotic consumption in populations with very low baseline infection risk [21]. In particular, concerns regarding antimicrobial resistance, *Clostridioides difficile* infection, and unintended downstream effects on the microbiome warrant careful consideration [22,23]. Additionally, standardized postoperative regimens may not account for patient-specific or institution-specific pathogen profiles, raising the possibility of a mismatch between antibiotic selection and infection risk. Our findings suggest that when baseline PJI rates are well below 1%, the absolute clinical benefit of EOA is minimal, resulting in a high number needed to treat that is unlikely to justify universal implementation. Rather than supporting routine adoption, these data favor a selective, risk-based approach to EOA, targeting patients or environments with elevated infection risk, while maintaining alignment with antibiotic stewardship goals. Further prospective studies incorporating microbiologic outcomes, resistance patterns, and adverse event monitoring are warranted to more fully define the risk–benefit profile of EOA.

### Limitations

This study has several limitations. First, its retrospective design introduces potential residual confounding despite covariate adjustment. The intervention was introduced in March 2023, meaning the study did not isolate the effect of EOA itself. In a maturing ASC pathway, changes in patient selection, workflow efficiency, discharge processes, surveillance intensity, and other unmeasured aspects of perioperative care may have evolved over time even if the formal infection–prevention protocol remained nominally unchanged. Under this design, the observed differences cannot be confidently attributed to EOA alone. Given the very low baseline infection rates, the observed differences may simply reflect random variation or other unmeasured factors associated with the retrospective study design rather than a true treatment effect. Additionally, PJI remains a rare event, contributing to statistical imprecision; longer follow-up is needed to assess the durability of the benefit fully. No temporal trend toward reduced infection was observed, independent of EOA adoption. Our center has strict glycemic thresholds, decolonization protocols, and standardized perioperative prophylaxis; the remaining infection risk may already be too low for EOA to provide clinically meaningful added value. Therefore, this study may be measuring the marginal effect of cefdinir on top of an already highly optimized system rather than the broader value of EOA as a prophylactic strategy. We effectively tested only one EOA protocol, as other EOA protocols could vary. The pharmacy acquisition cost was based on internal estimates and may vary across institutions and markets. Microbiologic resistance patterns and *Clostridioides difficile* risk were not assessed and warrant further investigation. Lastly, local antibiograms may influence the success of any EOA program, and the results from our local antibiogram may not apply to other local antibiograms.

## Conclusions

Although absolute benefit was limited in our low-risk ASC environment, reflected by high NNT estimates, the modeling suggests that EOA may become increasingly advantageous as baseline infection rates approach 1% or higher. Cost analyses demonstrated procedure-specific differences: EOA were frequently cost-effective and became cost-saving in TKA when the downstream cost of PJI exceeded approximately $13,300, whereas no cost-saving threshold was identified for THA within the evaluated range. Taken together, these findings do not support the routine use of universal EOA following standard-risk primary THA and TKA performed in low-risk ambulatory surgery centers. Instead, EOA may be most appropriately reserved for patients or practice settings with elevated baseline infection risk, consistent with prior literature demonstrating benefit in high-risk primary and aseptic revision arthroplasty. Prospective studies are warranted to further refine patient selection and confirm these findings across diverse practice environments.

## CRediT authorship contribution statement

**Breydan H. Wright:** Writing – review & editing, Writing – original draft, Supervision, Methodology. **Charlotte C. Baker:** Writing – review & editing, Writing – original draft, Software, Project administration, Investigation, Formal analysis. **Zachary M. Ricciardelli:** Writing – review & editing, Writing – original draft, Project administration, Investigation, Formal analysis. **Joseph M. Schwab:** Writing – review & editing, Supervision, Methodology. **Thomas L. Bradbury:** Writing – review & editing, Supervision, Methodology. **Courtney Levit:** Writing – review & editing, Project administration. **George N. Guild:** Writing – review & editing, Supervision, Resources, Methodology, Conceptualization.

## Conflicts of interest

Thomas L Bradbury receives royalties from Zimmer Biomet and Total Joint Orthopaedics; is a paid consultant for Zimmer Biomet and Total Joint Specialists; and serves as a board member for Georgia Orthopaedic Society. George N Guild is a paid consultant for Zimmer Biomet; holds stock or stock options in Total Joint Orthopaedics, Forcast; serves on the JOA editorial board; and serves as a Board member for Co-chair AAHKS FOCAL. Joseph M. Schwab receives honoraria for speakers bureau activities or paid presentations for DePuy Synthes; and serves as a Board member for Anterior Hip Foundation. All other authors declare no potential conflicts of interest.

For full disclosure statements refer to https://doi.org/10.1016/j.artd.2026.102108.

## Appendix A Supplementary data

Conflict of Interest Statement for Baker

Conflict of Interest Statement for Bradbury

Conflict of Interest Statement for Guild

Conflict of Interest Statement for Levit

Conflict of Interest Statement for Ricciardelli

Conflict of Interest Statement for Schwab

Conflict of Interest Statement for Wright

## References

[1] Shahi A., Tan T.L., Chen A.F., Maltenfort M.G., Parvizi J. (2017). In-hospital mortality in patients with periprosthetic joint infection. J Arthroplasty.

[2] Premkumar A., Kolin D.A., Farley K.X., Wilson J.M., McLawhorn A.S., Cross M.B. (2021). Projected economic burden of periprosthetic joint infection of the hip and knee in the United States. J Arthroplasty.

[3] Fischbacher A., Borens O. (2019). Prosthetic-joint infections: mortality over the last 10 years. J Bone Jt Infect.

[4] Schwartz A.M., Farley K.X., Guild G.N., Bradbury T.L. (2020). Projections and epidemiology of revision hip and knee arthroplasty in the United States to 2030. J Arthroplasty.

[5] Hegde V., Bracey D.N., Johnson R.M., Dennis D.A., Jennings J.M. (2021). Increased prevalence of depressive symptoms in patients undergoing revision for periprosthetic joint infection. Arthroplast Today.

[6] Berend K.R., Lombardi A.V., Morris M.J., Bergeson A.G., Adams J.B., Sneller M.A. (2013). Two-stage treatment of hip periprosthetic joint infection is associated with a high rate of infection control but high mortality. Clin Orthop.

[7] Natsuhara K.M., Shelton T.J., Meehan J.P., Lum Z.C. (2019). Mortality during total hip periprosthetic joint infection. J Arthroplasty.

[8] Zmistowski B., Karam J.A., Durinka J.B., Casper D.S., Parvizi J. (2013). Periprosthetic joint infection increases the risk of one-year mortality. J Bone Joint Surg Am.

[9] Kurtz S.M., Lau E.C., Son M.-S., Chang E.T., Zimmerli W., Parvizi J. (2018). Are we winning or losing the battle with periprosthetic joint infection: trends in periprosthetic joint infection and mortality risk for the medicare population. J Arthroplasty.

[10] Bukowski B.R., Owen A.R., Turner T.W., Fruth K.M., Osmon D.R., Pagnano M.W. (2022). Extended oral antibiotic prophylaxis after aseptic revision TKA: does it decrease infection risk?. J Arthroplasty.

[11] Bundschuh K.E., Muffly B.T., Ayeni A.M., Heo K.Y., Khawaja S.R., Tocio A.J. (2024). Should all patients receive extended oral antibiotic prophylaxis? Defining its role in patients undergoing primary and aseptic revision total joint arthroplasty. J Arthroplasty.

[12] Kheir M.M., Dilley J.E., Ziemba-Davis M., Meneghini R.M. (2021). The AAHKS clinical research award: extended oral antibiotics prevent periprosthetic joint infection in high-risk cases: 3855 patients with 1-year follow-up. J Arthroplasty.

[13] Mirahmadi A., Hosseini-Monfared P., Farrokhi M., Minaie R., Bateni A., Kazemi S.M. (2025). Extended oral antibiotics fail to reduce surgical site infection in orthopedic surgeries: a comparative study. PLoS One.

[14] Fuqua A.A., Worden J.A., Ayeni A.M., Bundschuh K.E., Premkumar A., Wilson J.M. (2025). Extended oral antibiotic prophylaxis and PJI-free survivorship after primary total knee arthroplasty. Knee.

[15] Lipson S., Pagani N.R., Moverman M.A., Puzzitiello R.N., Menendez M.E., Smith E.L. (2022). The cost-effectiveness of extended oral antibiotic prophylaxis for infection prevention after total joint arthroplasty in high-risk patients. J Arthroplasty.

[16] Parvizi J., Gehrke T., Chen A.F. (2013). Proceedings of the international consensus on periprosthetic joint infection. Bone Joint J.

[17] Goodman S.M., Springer B.D., Chen A.F., Davis M., Fernandez D.R., Figgie M. (2022). 2022 American College of Rheumatology/American Association of Hip and Knee Surgeons Guideline for the perioperative management of antirheumatic medication in patients with rheumatic diseases undergoing elective total hip or total knee arthroplasty. J Arthroplasty.

[18] Courtney P.M., Boniello A.J., Berger R.A. (2017). Complications following outpatient total joint arthroplasty: an analysis of a national database. J Arthroplasty.

[19] Inabathula A., Dilley J.E., Ziemba-Davis M., Warth L.C., Azzam K.A., Ireland P.H. (2018). Extended oral antibiotic prophylaxis in high-risk patients substantially reduces primary total hip and knee arthroplasty 90-day infection rate. J Bone Joint Surg Am.

[20] Berríos-Torres S.I., Umscheid C.A., Bratzler D.W., Leas B., Stone E.C., Kelz R.R. (2017). Centers for Disease Control and Prevention Guideline for the prevention of surgical site infection, 2017. JAMA Surg.

[21] (2018). Global guidelines for the prevention of surgical site infection.

[22] Ravi S., Zhu M., Luey C., Young S.W. (2016). Antibiotic resistance in early periprosthetic joint infection. ANZ J Surg.

[23] Privitera G., Scarpellini P., Ortisi G., Nicastro G., Nicolin R., de Lalla F. (1991). Prospective study of Clostridium difficile intestinal colonization and disease following single-dose antibiotic prophylaxis in surgery. Antimicrob Agents Chemother.

